# The complete chloroplast genome sequence of *Hordeum distichon* (Poales: Poaceae)

**DOI:** 10.1080/23802359.2021.2003260

**Published:** 2021-11-23

**Authors:** Xinlian Yu, Xin Li, Xiaohua Yao, Youhua Yao, Yixiong Bai, Likun An, Kunlun Wu

**Affiliations:** a Academy of Agricultural and Forestry Sciences, Qinghai University, Xining, Qinghai, China; b Qinghai Key Laboratory of Hulless Barley Genetics and Breeding, Xining, Qinghai, China; c Qinghai Subcenter of National Hulless Barley Improvement, Xining, Qinghai, China

**Keywords:** *Hordeum distichon*, chloroplast genome, phylogenetic relationship, *Triticeae*

## Abstract

*Hordeum distichon* (*H. distichon*) is a two-row cultivated barley used as food and as a feed crop. Chloroplast genome is an excellent way to study the genetic structure and evolutionary process of natural population of plant species in recent years. In this study, the complete chloroplast genome of *H. distichon* was sequenced and analyzed: the size of the chloroplast genome is 136,462 bp in length, including a large single copy region (LSC) of 80,597 bp, a small single copy region (SSC) of 12,701 bp, and a pair of inverted repeated regions (IR) of 21,582 bp; the *H. distichon* chloroplast genome encodes 129 genes, including 83 protein-coding genes, 38 tRNA genes, and eight rRNA genes; the overall GC-content of the chloroplast genome was 38.32%, with the LSC, SSC, and IR regions being 36.31%, 32.33%, and 43.83%, respectively. Phylogenetic analysis based on 32 species with the maximum likelihood (ML) method indicated that *H. distichon* was closely related to *Hordeum vulgare*.

*Hordeum distichon* (*H. distichon*), commonly known as two-row cultivated barley, belongs to the genus *Hordeum* of the family Poaceae which is the important cereal crop grown in nearly all the cultivated areas of the world (Carpici and Celik 2012). *H. distichon* is used for malting, and the best malt quality for beer is produced from two-row cultivated varieties (Bizuneh and Abebe 2019). Golden Promise is considered as a two-row cultivated barley variety of *H. distichon*, which is widely used in brewery barley breeding because of its high yield and excellent brewery quality (Schreiber et al. 2019; Reichel et al. 2021).

The chloroplast genome is smaller than the nuclear genome, and compared with mitochondrial genome, chloroplast genome structure is relatively conservative (Niu et al. 2020). Therefore, chloroplast genome is an excellent way to study the genetic structure and evolutionary process of natural population of plant species in recent years. Chloroplast genome has been widely used in plant research (Su et al. 2020).

In this study, the complete chloroplast genome of *H. distichon* was sequenced and analyzed by using the two-row cultivated barley variety Golden Promise as the research material. The fresh leaves of Golden Promise were collected from Qinghai University artificial climate incubator in Xining, Qinghai, China (101°78′ E, 36°62′ N) and the DNA of Golden Promise was deposited at the Qinghai Key Laboratory of Hulless Barley Genetics and Breeding (Xin Li, lixinyynq@163.com) under the storage number HG-20201230-01. The genomic DNA was extracted following the modified cetyl-trimethylammonium bromide (CTAB) from the leaf tissues (Doyle and Doyle 1987), and the genomic library was constructed via the VAHTS Universal DNA Library Prep Kit for Illumina V3 (Vazyme Biotech Co., Ltd, Nanjing, China). The chloroplast genome of *H. distichon* was sequenced on the Illumina Novaseq Platform (Illumina, San Diego, CA, USA) at Genepioneer Biotechnologies Inc., Nanjing, China. The high-quality reads (Clean Data) was obtained via fastp (v0.20.0) software (Chen et al. 2018), the Clean Data was assembled using SPAdes v3.10.1 (Bankevich et al. 2012) and annotated using prodigal v2.6.3 (https://www.github.com/hyattpd/Prodigal), hmmer v3.1b2 (http://www.hmmer.org/）and aragorn v1.2.38 (http://130.235.244.92/ARAGORN/) (Laslett and Canback 2004; Hyatt et al. 2010; Mistry 2013), and manually correct the annotations with another result obtained by blast v2.6 (https://blast.ncbi.nlm.nih.gov/Blast.cgi) (Altschul 1990).

The size of the chloroplast genome is 136,462 bp in length, including a large single copy region (LSC) of 80,597 bp, a small single copy region (SSC) of 12,701 bp, and a pair of inverted repeated regions (IR) of 21,582 bp. And the *H. distichon* chloroplast genome encodes 129 genes, including 83 protein-coding genes, 38 tRNA genes, and eight rRNA genes. The overall GC-content of the chloroplast genome was 38.32%, with the LSC, SSC, and IR regions being 36.31, 32.33, and 43.83%, respectively.

Thirty species in 17 genera of *Triticeae* and an additional outgroup species (*Oryza sativa*) were selected from the National Center for Biotechnology Information (NCBI) for investigating the phylogeny of *H. distichon* (Genbank: MW531731.1). Alignment was conducted using MAFFT software (Katoh and Standley 2013). The phylogenetic tree was built using the MEGA 7 software (http://www.megasoftware.net/mega.html) with the maximum likelihood (ML) method and the bootstrap set to 1,000 (Kumar et al. 2016; Felsenstein 1985). The phylogenetic tree analysis strongly supported that *H. distichon* was closely relate to *Hordeum vulgare* (Figure 1). This study will provide the genome and genetic resources for the further study of *H. distichon*, and also lay the foundation for the study of chloroplast genome engineering of the *H. distichon*.

**Figure 1.**
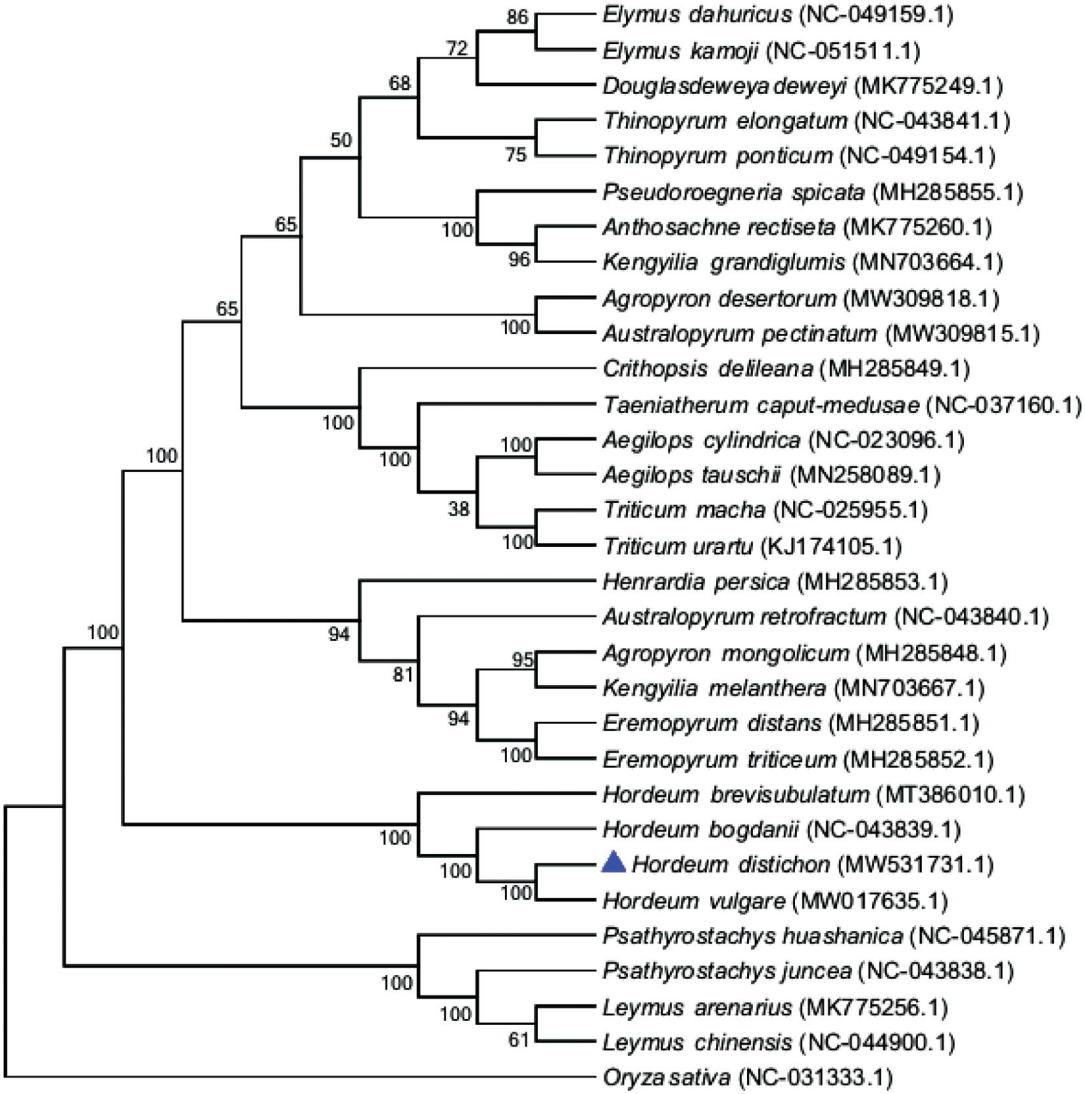
Phylogenetic tree based on the chloroplast genome of 30 species in 17 genera of *Triticeae* and taking *Oryza sativa* as an outgroup.

## Data Availability

The genome sequence data that support the findings of this study are openly available in GenBank of NCBI at (https://www.ncbi.nlm.nih.gov/) under the accession no. MW531731.1; the associated BioProject, SRA, and Bio-Sample numbers are PRJNA743923, SRR15044534, and SAMN20063185, respectively.
